# Multiple Self-Made Side Holes in a Fully Covered Metal Stent Prevent Intrahepatic Bile Duct Occlusion Following Endoscopic Ultrasound-Guided Hepaticogastrostomy: A Retrospective Study in Japan

**DOI:** 10.3390/jcm14113773

**Published:** 2025-05-28

**Authors:** Ren Kuwabara, Kazuo Hara, Shin Haba, Takamichi Kuwahara, Nozomi Okuno, Hiroki Koda, Minako Urata, Takashi Kondo, Yoshitaro Yamamoto, Keigo Oshiro, Tomoki Ogata

**Affiliations:** Department of Gastroenterology, Aichi Cancer Center Hospital, 1-1 Kanokoden, Chikusa-ku, Nagoya 464-8681, Japan

**Keywords:** drainage, cholangitis, endoscopy, endoscopic ultrasound-guided hepaticogastrostomy, fully covered self-expandable metal stent

## Abstract

**Background/Objectives**: Endoscopic ultrasound-guided hepaticogastrostomy (EUS-HGS) using a fully covered self-expandable metal stent (FCSEMS) is an alternative to endoscopic retrograde cholangiopancreatography for biliary drainage; however, FCSEMSs may cause intrahepatic bile duct (IHD) obstruction and cholangitis. In this study, we developed an FCSEMS with multiple self-made side holes at its tip and evaluated its safety and efficacy. **Methods**: This retrospective study included 100 patients who underwent EUS-HGS with FCSEMS placement between April 2022 and October 2023. Fifty patients received a conventional FCSEMS, and 50 received an FCSEMS with multiple self-made side holes. Technical and clinical success, residual contrast in the IHD, recurrent biliary obstruction (RBO), and adverse events (AEs) were then evaluated. The clinical success rates were 98% and 90% for the side hole and conventional FCEMS groups, respectively. The amount of residual contrast in the IHD was lower in the side hole group (0% vs. 12%, *p* = 0.027). RBO incidence was significantly lower in the side hole group (8% vs. 30%, *p* < 0.001), with migration as the primary cause in the conventional group. Early AEs, including segmental cholangitis, occurred only in the conventional group. During reintervention, all stents were safely removed. **Conclusions**: The FCSEMSs with multiple side holes reduced IHD occlusion and cholangitis, improving biliary drainage and safety. Further studies are needed to confirm these findings.

## 1. Introduction

Biliary drainage is an essential therapeutic strategy for patients with obstructive jaundice, particularly those with malignant biliary obstruction. The first-line approach for biliary drainage is typically endoscopic retrograde cholangiopancreatography (ERCP). However, ERCP can be unsuccessful in cases with altered surgical anatomy, tumor infiltration of the papilla, or duodenal obstruction. In such situations, alternative drainage routes become necessary.

Endoscopic ultrasound-guided biliary drainage (EUS-BD) has gained increasing attention as a minimally invasive alternative to percutaneous transhepatic biliary drainage (PTBD), which has traditionally been used in ERCP-failed cases. Among various EUS-BD techniques, endoscopic ultrasound-guided hepaticogastrostomy (EUS-HGS) is particularly useful when access to the left intrahepatic bile ducts (segments B2 or B3) is possible [1]. EUS-HGS involves the creation of a fistula between the stomach and the intrahepatic bile duct using a stent to allow internal drainage.

EUS-HGS can be performed using either self-expandable metal stents (SEMSs) or plastic stents (PSs). Compared to PSs, SEMSs provide superior patency, lower occlusion rates, and greater long-term durability [2]. Fully covered SEMSs (FCSEMSs) are particularly effective in preventing bile leakage [3]; however, their use may result in obstruction of the intrahepatic bile duct (IHD), especially in branched segments, potentially leading to segmental cholangitis or insufficient biliary drainage.

Residual contrast medium is sometimes observed in the IHD on computed tomography (CT) the day after EUS-HGS. Partially covered SEMSs have recently been introduced to address some of these limitations [4]. Although these stents allow for endoscopic reintervention through their lumen [5], they are difficult to remove due to tissue ingrowth and hyperplasia.

To overcome these challenges, we developed a novel technique to create multiple self-made side holes at the tip of a conventional FCSEMS. In this study, we retrospectively evaluated the safety and efficacy of this modified stent design for preventing IHD obstruction and improving clinical outcomes in patients undergoing EUS-HGS.

## 2. Materials and Methods

### 2.1. Study Design

This study was a single-center, retrospective study conducted at the Aichi Cancer Center Hospital, Japan. Patients were enrolled consecutively during the study period. The stent type was assigned according to a fixed institutional timeline, not physician preference. One hundred consecutive patients underwent EUS-HGS with an FCSEMS (6 mm × 12 cm HANAROSTENT Benefit; Boston Scientific, Natick, MA, USA) between April 2022 and October 2023. The first 50 patients, treated between April to December 2022, received an FCSEMS without side holes. The subsequent 50 patients, treated between January and October 2023, received an FCSEMS with multiple self-made 15 mm long side holes. All patients provided informed consent for the procedure. The study was approved by the institutional review board of Aichi Cancer Center (approval number: IR061183) and was conducted in accordance with the Declaration of Helsinki.

### 2.2. EUS-HGS Procedure

All patients were placed under conscious intravenous sedation during the procedure and received intravenous prophylactic antibiotics. EUS was performed using an oblique-viewing echoendoscope (GF-UCT260; Olympus, Tokyo, Japan, or EG-740UT; FUJIFILM Medical, Tokyo, Japan) or forward-viewing EUS (TGF-UCT260J; Olympus). First, a landmark clip was placed at the esophagogastric junction to enable easy identification under fluoroscopic guidance and prevent esophagus puncture [6]. A 22-gauge needle (Expect Slimline; Boston Scientific) was preloaded with a 0.018-inch guidewire (Fielder 18; Olympus) through the connector (Rotating Hemostatic Valve 0.096 Abbott, Tokyo, Japan) and filled with contrast medium. A 19-gauge needle (EZ Shot 3 Plus; Olympus) was preloaded with a 0.025-inch guidewire (M-through; Asahi Intecc, Aichi, Japan, or VisiGlide 2; Olympus) through the connector (Radifocus Hemostasis Valve II; Terumo, Tokyo, Japan) and filled with contrast medium. Color Doppler ultrasound guidance was used to avoid intervening vessels when the IHDs were punctured. We inserted a guidewire into the B2 or B3 IHDs and subsequently injected a small amount of contrast medium. In some cases, the needle tract was gradually dilated using a drill dilator (Tornus ES; Asahi Intecc, Aichi, Japan), tapered tip cannula (Uneven Double Lumen Cannula; PIOLAX or ES Dilator soft type; Zeon Medical, Tokyo, Japan or EndoSheather; PIOLAX, Kanagawa, Japan), and balloon catheter (REN; KANEKA, Osaka, Japan). We then exchanged the biliary catheter (Tandem XL Triple Lumen ERCP Cannula; Boston Scientific or Uneven Double Lumen Cannula; PIOLAX) to inject contrast medium and aspirate the bile juice. Subsequently, an FCSEMS (6 mm × 12 cm HANAROSTENT Benefit; Boston Scientific) was placed [7].

**Figure 1:**
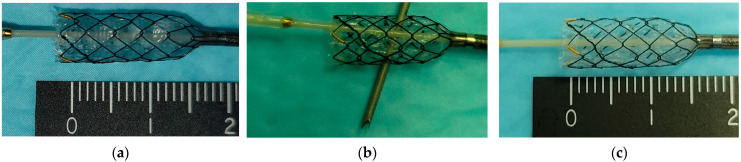
How to create multiple side holes. (**a**) A fully covered self-expandable metal stent was pulled out 15 mm from the delivery system. (**b**) Each mesh was punctured with a 19-gauge needle (Acquire; Boston Scientific). (**c**) Multiple side holes were created at 15 mm from the stent tip.

From January 2023, an FCSEMS with multiple self-made side holes was used. We then measured the length from the B2–B3 confluence to the punctured IHD point on a fluoroscopic image as 15 mm. An FCSEMS was pulled out 15 mm from the delivery system before insertion into the scope, and multiple small side holes were created in each mesh space using a 19-gauge needle (Acquire; Boston Scientific) (Figure 1) and re-sheathed completely. The FCSEMS with multiple self-made side holes was placed with the proximal tip located at the B3 junction site so that the side holes aligned with the IHD area [8]. Finally, a clip was placed on the gastric side [9].

### 2.3. Definitions

**Figure 2:**
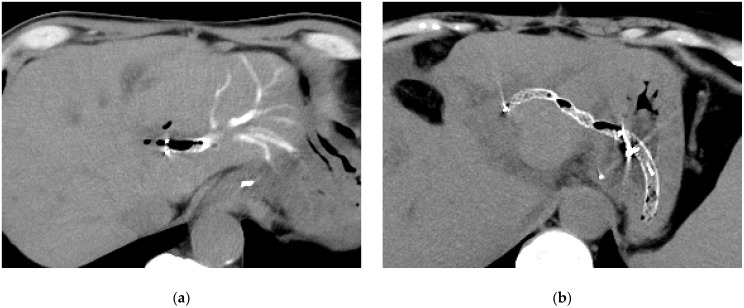
Computed tomography image taken the day after endoscopic ultrasound-guided hepaticogastrostomy (EUS-HGS). (**a**) The contrast medium remains in the bile duct beyond the HGS stent placement site. (**b**) There is no residual contrast medium in the bile duct.

We defined technical success as successful stent placement and clinical success as achieving either normalization of the serum total bilirubin level or less than 50% of the pre-drainage level 14 days post-procedure. Residual contrast medium (Figure 2) and bile leakage were evaluated using CT images obtained the day after the procedure. Recurrent biliary obstruction (RBO) was identified when cholangitis or jaundice was present, with imaging confirming biliary dilatation. Adverse events (AEs) occurring after the procedure and potentially related to it were classified according to the American Society for Gastrointestinal Endoscopy lexicon [10]. Acute cholangitis and cholecystitis were classified according to the criteria reported in the 2018 Tokyo Guidelines [11]. A fistula break was defined as disruption of the fistula during reintervention.

### 2.4. Statistical Analysis

Continuous variables were analyzed using *t*-tests. Categorical variables were analyzed using Fisher’s exact test or the chi-square test. The Log-rank test was used to compare the time to RBO (TRBO) between groups. The Kaplan–Meier method was used to estimate the TRBO. All statistical analyses were performed using EZR software (Saitama Medical Center, Jichi Medical University, Saitama, Japan).

## 3. Results

The FCSEMSs with multiple self-made side holes were placed in 50 patients (median age, 65.3 years; age range, 36–91 years; 30 males); similarly, FCSEMSs without side holes were also used in 50 other patients (median age, 64.3 years; age range, 24–82 years; 25 males). The patient characteristics are summarized in Table 1.

### 3.1. Outcomes of EUS-HGS

Stent placement was unsuccessful in only one patient who received a conventional FCSEMS. The clinical success rates were 98% (49/50) and 90% (45/50) for the side hole and conventional FCSEMS groups, respectively. The amount of residual contrast medium was significantly lower in the side hole group than in the conventional FCSEMS group (side hole group, 0 (0%); conventional FCSEMS group, 6 (12%); *p* = 0.027). The number of RBO events in the side hole group was lower than that in the conventional FCSEMS group (side hole group, 4 (8%); conventional FCSEMS group, 15 (30%); *p* < 0.001). Stent occlusion explained the development of RBO in the side hole group; whereas RBO developed due to stent occlusion in three patients (6%) and stent migration in 12 patients (24%) in the conventional FCSEMS group. The outcomes of EUS-HGS are summarized in Table 2.

### 3.2. Adverse Events

**Figure 3:**
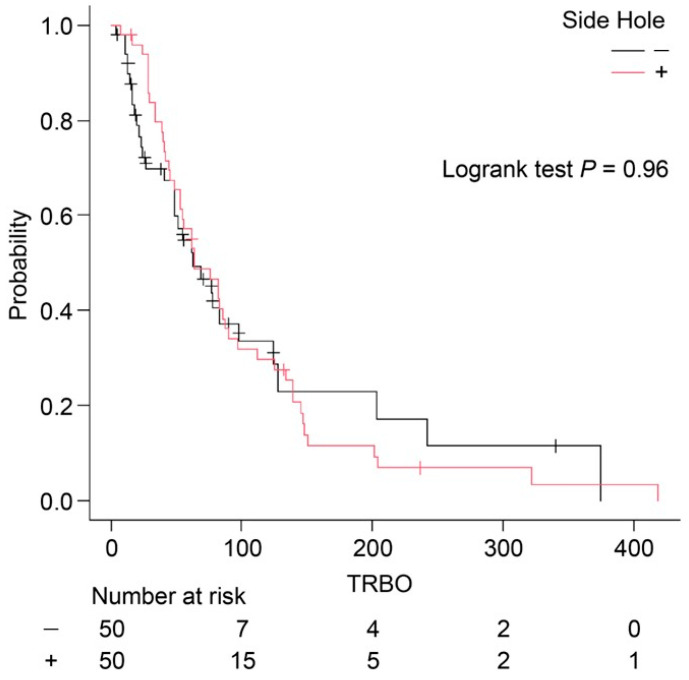
Kaplan–Meier curves for the time to recurrent biliary obstruction (TRBO). The TRBO of the fully covered self-expandable metal stent (FCSEMS) with multiple self-made side holes (red line; median 64 days; 95% confidence interval (CI): 53–87) was not significantly different from the TRBO of the conventional FCSEMS (black line; median 63 days; 95% CI: 49–98) (*p* = 0.96).

Among the early AEs, segmental cholangitis (mild, *n* = 2; moderate, *n* = 1) occurred only in the conventional FCSEMS group. Bile leakage and peritonitis were not observed during the follow-up period. Although the incidence of RBO was lower in the side hole group, there was no significant difference in the TRBO between the two groups (side hole group: median TRBO, 64 days; conventional FCSEMS group: median TRBO, 63 days; *p* = 0.96) (Figure 3). This apparent discrepancy—lower RBO incidence despite a similar median TRBO—may be attributed to the distribution pattern of RBO events and is discussed further in the Section 4. A summary of AEs associated with EUS-HGS is provided in Table 3.

### 3.3. Reintervention

Reintervention was required in 36 patients in the side hole group (72%) and 32 in the conventional FCSEMS group (64%), and was scheduled in 32 patients in the side hole group (89%) and 15 in the conventional FCSEMS group (47%). In the side hole group, reintervention required stent exchange. In the conventional FCSEMS group, eighteen patients (56%) required stent exchange, seven patients (22%) required re-stenting after distal stent migration, five patients (16%) required reattempted HGS, and two patients (6%) required additional stenting in other regions. In all patients, the reintervention success rate was 100% (68/68). All stents were safely removed without complications. In the conventional FCSEMS group, fistula breaks occurred during reintervention (*n* = 2). No other AEs were observed during the follow-up. The details of reintervention are summarized in Table 4.

## 4. Discussion

In this study, we investigated the potential benefits of creating multiple self-made side holes at the tip of an FCSEMS to prevent IHD occlusion following EUS-HGS. Conventional FCSEMSs, while effective in reducing bile leakage, often lead to IHD obstruction due to their fully covered structure, which can result in segmental cholangitis and inadequate biliary drainage [12]. Our findings suggest that multiple self-made side holes can alleviate this issue.

Post-procedural CT revealed no residual contrast medium in the IHD in the side hole group, whereas 12% of the patients in the conventional FCSEMS group exhibited contrast retention (*p* = 0.027).

The incidence of RBO was significantly lower in the side hole group (8%) than in the conventional FCSEMS group (30%) (*p* < 0.001). Side holes may enhance bile drainage, reducing the risk of bile stasis and subsequent infection. It is also possible that the creation of side holes rendered the stents less likely to migrate.

The reintervention success rate was 100% in both groups. Furthermore, TRBO was not significantly different between the two groups (64 vs. 63 days, *p* = 0.96). Although the incidence of RBO was lower in the side hole group, the median TRBO was nearly identical between the two groups. As shown in the Kaplan–Meier curves, both groups exhibited a rapid decline in patency within the first 100 days, after which the curves plateaued. This suggests that most RBO events occurred early in the clinical course, regardless of the stent type, resulting in similar median TRBO values (64 vs. 63 days).

However, a closer examination of the number at risk and the tails of the curves indicates a trend toward longer-term patency in a small subset of patients in the side hole group. Despite this, the overall impact on median TRBO was minimal, as the early events strongly influenced the median. This may explain why a lower overall RBO incidence did not translate into a longer TRBO. Furthermore, the log-rank test (*p* = 0.96) supports the absence of a statistically significant difference in stent patency over time.

These findings suggest that while the addition of side holes may contribute to a reduction in RBO events, particularly in specific anatomical or clinical scenarios, this benefit may not be fully captured by median TRBO analysis alone. Additional metrics such as event-free survival rate at fixed time points (e.g., 30, 90, and 180 days) or cumulative incidence of RBO could provide a more nuanced evaluation of stent performance. Future prospective studies with stratified analyses are warranted to clarify the potential benefit of side hole modifications.

The proposed mechanism behind the benefit of side holes involves the facilitation of bile flow from adjacent intrahepatic duct branches that might otherwise be blocked by the fully covered membrane of the stent. By creating multiple small fenestrations at the proximal stent portion, the stent maintains communication with nearby ducts even when its main axis does not align perfectly with the biliary anatomy. This may help prevent bile stagnation, reducing the risk of localized infection and segmental cholangitis. Although direct imaging or flow simulation data were not available in this study, the absence of residual contrast medium and lower RBO incidence support this physiological hypothesis.

These findings highlight a simple yet potentially impactful technical modification that can be applied to existing FCSEMS devices without requiring specialized materials or devices. Given the increasing use of EUS-HGS in patients with complex or advanced malignancies, optimizing stent performance while maintaining removability is of considerable clinical importance. Furthermore, avoiding stent migration—frequently observed in the conventional FCSEMS group—may reduce the need for emergency reinterventions and improve patient safety. Efforts to standardize the method for creating side holes and develop pre-manufactured stents with this feature may further improve generalizability and facilitate broader clinical adoption.

This study was limited by its retrospective design, relatively small sample size, and single-center setting. Future prospective, multicenter trials are needed to validate these findings and explore whether specific patient subgroups derive particular benefit from the side hole modification. Additionally, computational modeling or in vitro flow studies may help elucidate the detailed fluid dynamics enabled by this approach.

## 5. Conclusions

Our findings suggest that creating multiple self-made side holes at the tip of the FCSEMS can reduce IHD occlusion and segmental cholangitis. Moreover, the side holes may have made the stent less likely to migrate after EUS-HGS. An FCSEMS with multiple self-made side holes may enhance the safety of reintervention. Additionally, the development of a standardized and simplified method for creating side holes may facilitate wider clinical application and improve patient outcomes.

## Figures and Tables

**Table 1:** Patient characteristics.

	Side Hole Group (*n* = 50)	Conventional FCSEMS Group (*n* = 50)	*p*-Value
Age, median (range), years	65.3 (36–91)	64.3 (24–82)	0.962 ^a^
Sex, male, *n* (%)	30 (60)	25 (50)	0.422 ^b^
ECOG performance status (0/1/2/3), *n* (%)	38 (76)/11 (22)/1(2)/0 (0)	26 (52)/16 (32)/6 (12)/2 (4)	**0.0276** ^b^
Causes of biliary stricture, *n* (%)			0.166 ^c^
Pancreatic cancer	32 (64)	17 (34)	
Gallbladder cancer	4 (8)	6 (12)	
Bile duct cancer	4 (8)	9 (18)	
Hepatocellular carcinoma	1 (2)	2 (4)	
Gastric cancer	1 (2)	5 (10)	
Duodenal cancer	1 (2)	2 (4)	
Sarcoma	1 (2)	1 (2)	
Distant metastasis	4 (8)	3 (6)	
Benign biliary stricture	2 (4)	5 (10)	
Ascites, mild/moderate, *n* (%)	13 (26)/3 (66)	12 (24)/8 (16)	0.291 ^b^
Anticoagulant therapy, *n* (%)	2 (4)	2 (4)	1 ^b^
Indication for EUS-HGS, *n* (%)			0.857 ^c^
Primary drainage	31 (62)	26 (52)	
Salvage drainage	7 (14)	11 (22)	
Surgically altered anatomy	7 (14)	7 (14)	
Combination with ERCP	4 (8)	5 (10)	
Conversion from ERCP	1 (2)	1 (2)	
Diameter of the punctured IHD, mean(range), mm	3.3 (0.9–7.6)	3.9 (1.0–10.6)	0.126 ^a^
Puncture site, *n* (%)			0.176 ^b^
B2	40 (80)	33 (66)	
B3	10 (20)	17 (34)	
Needle gauge, *n* (%)			0.208 ^b^
19 G	21 (42)	33 (66)	
22 G	29 (58)	17 (34)	

ECOG, Eastern Cooperative Oncology Group; EUS-HGS, Endoscopic Ultrasound-guided Hepaticogastrostomy; ERCP, endoscopic retrograde cholangiopancreatography; IHD, intrahepatic bile duct; ^a^ *t*-test; ^b^ Fisher’s exact test; ^c^ Chi-square test.

**Table 2:** Outcomes of EUS-HGS.

	Side Hole Group (*n* = 50)	Conventional FCSEMS Group (*n* = 50)	*p*-Value
Technical success rate, *n* (%)	50 (100)	49 (98)	1.000 ^a^
Clinical success rate, *n* (%)	49 (98)	45 (90)	0.204 ^a^
Residual contrast medium in the branched intrahepatic bile duct, *n* (%)	0 (0)	6 (12)	**0.027 ^a^**
RBO, *n* (%)	4 (8)	15 (30)	**<0.001 ^b^**
Occlusion	4	3	1 ^b^
Migration	0	12	**<0.001 ^b^**

EUS-HGS, endoscopic ultrasound-guided hepaticogastrostomy; RBO, recurrent biliary obstruction; ^a^ Fisher’s exact test; ^b^ *t*-test.

**Table 3:** Adverse events following EUS-HGS.

	Side Hole Group (*n* = 50)	Conventional FCSEMS Group (*n* = 50)	*p*-Value ^a^
Early adverse events (≦14 days), *n*	1	4	0.179
Bleeding (mild)	1	0	1
Segmental cholangitis (mild/moderate)	0	2/1	0.242
Bile leakage	0	0	n.s.
Late adverse events (>14 days), *n*	4	3	0.678
Cholangitis (mild)	4	2	0.674
Cholecystitis	0	1	1
Bile leakage	0	0	n.s.
Peritonitis	0	0	n.s.

EUS-HGS, endoscopic ultrasound-guided hepaticogastrostomy; n.s., no significant difference; ^a^ Fisher’s exact test.

**Table 4:** Details of reintervention.

	Side Hole Group (*n* = 36)	Conventional FCSEMS Group (*n* = 32)	*p*-Value ^a^
Cases of reintervention, *n* (%)			**<0.001**
Scheduled reintervention	32 (89)	15 (47)	
Unexpected reintervention	4 (11)	17 (53)	
Contents of reintervention, *n* (%)			**<0.001**
Stent exchange	36 (100)	18 (56)	
Re-stenting after distal stent migration	0 (0)	7 (22)	
Reattempted HGS	0 (0)	5 (16)	
Additional stenting in other regions	0 (0)	2 (6)	
Success rate of reintervention, *n* (%)	36 (100)	32 (100)	1
Adverse events of reintervention, *n*	0	2	0.495
Fistula breaks	0	2	0.495
Bleeding	0	0	n.s.
Segmental cholangitis	0	0	n.s.
Bile leakage	0	0	n.s.
Peritonitis	0	0	n.s.

HGS, hepaticogastrostomy; n.s., no significant difference; ^a^ Fisher’s exact test.

## Data Availability

The data are not publicly available due to institutional restrictions.

## Abbreviations

ERCP	Endoscopic retrograde cholangiopancreatography
EUS-BD	Endoscopic ultrasound-guided biliary drainage
PTBD	Percutaneous transhepatic biliary drainage
EUS-HGS	Endoscopic ultrasound-guided hepaticogastrostomy
FCSEMS	Fully covered self-expandable metal stent
IHD	Intrahepatic bile duct
RBO	Recurrent biliary obstruction
TRBO	Time to recurrent biliary obstruction
AE	Adverse event
CT	Computed tomography
ERCP	Endoscopic retrograde cholangiopancreatography
